# Homocysteine—Potential Novel Diagnostic Indicator of Health and Disease in Horses

**DOI:** 10.3390/ani13081311

**Published:** 2023-04-11

**Authors:** Marcin Gołyński, Michał Metyk, Jagoda Ciszewska, Marcin Paweł Szczepanik, Gareth Fitch, Paweł Marek Bęczkowski

**Affiliations:** 1 Faculty of Biological and Veterinary Sciences, Nicolaus Copernicus University in Toruń, 87-100 Toruń, Poland; 2 Sub-Department of Diagnostics and Veterinary Dermatology, Faculty of Veterinary Medicine, University of Life Sciences in Lublin, 20-033 Lublin, Poland; 3 Department of Veterinary Clinical Sciences, Jockey Club College of Veterinary Medicine and Life Sciences, City University of Hong Kong, Kowloon Tong, Hong Kong, China

**Keywords:** homocysteine, equine, antioxidant, stress, surrogate marker

## Abstract

**Simple Summary:**

Homocysteine is an organic compound that can be measured in the blood of humans and animals. High levels of homocysteine in human blood are associated with an increased risk of heart disease, diseases of blood vessels, formation of blood clots and brain damage. However, the role of homocysteine in the health and disease of domestic animals is poorly understood. This review critically appraises the literature concerning homocysteine in animals, focusing on horses. It aims to clearly define the existing knowledge gap to path an avenue for future research into homocysteine as a potential diagnostic marker of health and disease in this species.

**Abstract:**

Homocysteine is an endogenous, non-protein sulfuric amino acid, an intermediate metabolite formed by the methionine transmethylation reaction. Its elevated serum concentration in humans, hyperhomocysteinemia, is a sensitive indicator and a risk factor for coagulation disorders, cardiovascular diseases and dementia. However, the role of homocysteine in veterinary species has not been unequivocally established. Although some research has been conducted in dogs, cats, cattle and pigs, relatively few studies on homocysteine have been conducted in horses. So far, it has been established in this species that homocysteine has an atherogenic effect, plays a role in early embryo mortality and is responsible for the induction of oxidative stress. These preliminary findings support establishing a reference range in a normal population of horses, including horses in training and merit further investigations into the role of this amino acid in health and disease in this species.

## 1. Introduction

**Figure 1:**
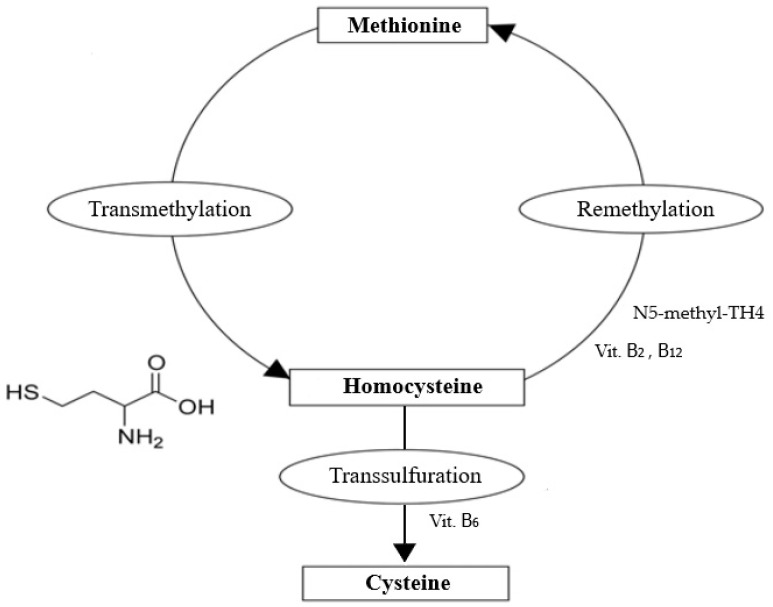
Schematic overview of homocysteine (Hcy) metabolism. Hcy is exclusively derived from dietary methionine. It can be catabolised via a transsulfuration pathway to cysteine or remethylated to methionine. Vitamins of the B-group (vit. B6; pyridoxine, vit. B2; riboflavin, vit. B12; cobalamin and biologically active form of vit. B9, N5-methyl-tetrahydrofolate (N5-methyl-TH4) play a crucial role in this pathway.

Homocysteine (Hcy) is an endogenous, non-proteinogenic, sulfur-containing amino acid formed as an intermediate metabolite in the process of intracellular transmethylation of an essential amino acid methionine to semi-essential cysteine (Figure 1) [1]. According to the latest hypothesis, homocysteine and its thiolactone could have been involved in protein synthesis at the origins of life on Earth [2]. Hcy is normally catabolized via the transsulfuration pathway to cysteine, but it can also be recycled back to methionine via (re)methylation [3,4]. The (re)methylation process takes place in the presence of a biologically active form of folic acid (vitamin B9), N5-methyl-tetrahydrofolate (N5-methyl-TH4) [5]. N5-methyl-TH4 is responsible for lowering Hcy concentrations, and its “sweeping” properties highlight the health benefit of dietary supplementation of folic acid [6,7,8,9,10]. A similar relationship exists with other nutrients. Methionine synthesis requires riboflavin (vitamin B2) and cobalamin (vitamin B12) as cofactors [11,12], whilst the transsulfuration of Hcy to cysteine requires pyridoxine (vitamin B6) [13]. Deficiencies of vitamins B2, B6 and B12 contribute to harmful hyperhomocysteinemia, and their supplementation reduces its effects [10,14,15,16,17,18,19,20,21,22,23].

In humans, increased serum Hcy concentration, hyperhomocysteinemia, is a sensitive marker and a risk factor for coagulation disorders, cardiovascular diseases, especially of thrombotic aetiology, neurodegenerative diseases and dementia [24,25,26,27,28,29,30,31,32]. Due to the coexistence of those medical conditions and hyperhomocysteinemia, particularly in older people, Hcy has been named by the medical profession as the cholesterol of the 21st century [33]. Recently, homocysteine has been investigated during COVID-19, chronic kidney disease, oncogenesis and infertility, emphasizing an interest in this biomarker within the medical and scientific community [34,35,36,37,38,39].

However, relatively little is known about the role of homocysteine in the health and disease of veterinary species. This review aims to critically appraise existing literature and clearly define the knowledge gap to pave the way for future research into serum homocysteine as a potential biomarker of health and disease in horses.

## 2. Methods

This paper is a narrative review. Research studies were ascertained by searching PubMed, The Web of Science, Google Scholar, and citation searching. Search terms included: homocysteine, AND horses OR equine OR pets OR dogs OR farm animals. The arbitrary start date for studies included in this review was not set. However, searches were completed in January 2023.

## 3. Homocysteine in Pets and Farm Animals

The role of hyperhomocysteinemia has not been clearly understood in veterinary medicine. However, a statistically significant increase in serum concentrations had been demonstrated in dogs with heart disease, dogs with chronic enteropathy, dogs and cats with kidney disease, and dogs with hypothyroidism [40,41,42,43].

During canine hypothyroidism, as in humans, the concentration of Hcy was negatively correlated with the concentration of total thyroxine (TT4) [42,44,45]. It is partially explained by the positive correlation between thyroid hormone levels and the activity of methylenetetrahydrofolate reductase (MTHFR), which is involved in the (re)methylation of Hcy to methionine [46,47,48,49]. This relationship indicates a likely role of thyroid gland disorders in developing homocysteine-dependent pathologies [44,50]. Therefore, any investigation of Hcy disturbances should ideally involve concurrent measurement of the thyroid hormone concentrations to assess their involvement.

Hcy has been studied in farm animals, but the evaluation of its serum concentration has not gained clinical application. The observations in pregnant and lactating sows provide interesting but somewhat ambiguous conclusions [51]. In a study assessing the effect of folic acid and vitamin B12 supplementation on the parameters of growth and the immune status of their offspring, there was a positive correlation between the concentration of Hcy and the growth performance of piglets, but on the other hand, a negative correlation with some indicators of humoral and cellular immune responses [51]. Furthermore, it has been demonstrated that a methionine-rich diet was correlated with serum hyperhomocysteinemia and predisposed pigs to atherogenesis [52]. Hcy has also been investigated in cattle [53,54,55,56]. Its serum concentration was significantly higher than in healthy animals during bovine theileriosis [53], and in moderate, long-term cobalt deficiency [54].

## 4. Homocysteine in Horses

The role of Hcy in disease pathogenesis has also been studied in horses, but to a limited extent and only in some areas in this species (Table 1).

### 4.1. Cardiovascular Disease

The role of Hcy in the etiology of cardiovascular disease in humans is well understood [64]. Hyperhomocysteinemia in humans has been associated with vascular inflammation and atherosclerosis [65,66,67,68,69,70,71,72]. Therefore, inhibition of vascular thromboresistance in hyperhomocysteinemia is of particular interest. Hcy increases the synthesis of thromboxane A2 (TxA2), activates coagulation factor V, inhibits the synthesis of anticoagulants at the DNA level, and suppresses the maturation of the endothelial matrix cells [73,74,75,76,77,78,79].

Furthermore, Hcy negatively affects the regeneration of already damaged vascular endothelial cells because it strongly inhibits DNA and protein methylation [80,81,82,83]. For example, Hcy inhibits the methylation of p21 Ras protein (p21^ras^) and decreases the expression of the gene encoding this protein [84]. This leads to a reduction in cellular DNA synthesis and impaired tissue repair [81]. Hcy also has a negative effect on myocytes and myocardial contractility [85].

The role of Hcy in horses in the course of laminitis is debatable. Although Hcy interacted with vascular endothelial cells in vitro, there was no association between hyperhomocysteinemia and the risk of laminitis [58].

However, a relationship between hyperhomocysteinemia, impaired chorionic angiogenesis and early embryo mortality was demonstrated in mares [57]. Therefore, determining serum Hcy concentration may be useful in assessing the risk of the above-mentioned fertility impairment in this species.

Because of its involvement in the proliferation of endothelial cells and vascular smooth muscle, Hcy affects the structure and function of the cardiovascular system. However, no relationship between hyperhomocysteinemia and cardiac dysfunction has yet been demonstrated in Equidae. In a study assessing horses with atrial fibrillation (AF), there were no differences in serum Hcy concentration between horses with AF (*n* = 55) and healthy animals (*n* = 27) [61]. Furthermore, there was no relationship between serum Hcy concentrations and the likelihood of AF recurrence after successful cardioversion [61].

In another study assessing serum Hcy, cardiac (troponin I, creatinine kinase and d-dimer) and renal biomarkers (urea, creatinine and cystatin-C) in horses naturally infected with *Theileria equi*, all examined parameters, including cardiac ones significantly positively correlated with the magnitude of parasitemia [62]. This may indicate the possible involvement of Hcy in myocardial damage, although other causes, including oxidative stress and the systemic response to parasitemia, are also possible.

Although based on small sample size, these observations emphasize a significant variation in the disease pathogenesis between horses and other species and highlight the differences in the potential utility of serum Hcy concentrations as a marker of cardiac disease in Equidae.

As aforementioned, more research is needed to further assess Hcy as a risk factor for vascular disease in horses.

### 4.2. Neurodegenerative Disease

Hyperhomocysteinemia in people leads to cognitive impairment and depression [86,87,88,89,90,91,92]. It is also a significant risk factor for Parkinson’s disease (PD) [93]. In a rat model of the disease, hyperhomocysteinemia reduces the number of dopaminergic neurons, likely by increasing their sensitivity to endogenous toxins [94,95]. In addition, Hcy contributes to neuronal degeneration by inducing oxidative stress, enhancing mitochondria dysfunction, DNA damage and apoptosis [96,97,98,99].

Amongst other neurodegenerative diseases, Alzheimer’s disease (AD), the most common cause of dementia in older adults, merits special consideration [100,101]. AD is primarily characterized by the deposition of the β-amyloid peptide (Aβ) in the brain parenchyma and cerebral blood vessels [102,103]. Although not entirely clear, hyperhomocysteinemia has been implicated in the pathogenesis of AD and other types of dementia [30,104,105,106,107,108,109]. It has also been demonstrated in a large meta-analysis that every increase of serum Hcy concentration by 5 µmol/L increases the risk of Alzheimer’s disease by as much as 12% [105]. It is fascinating that apart from being involved in vascular changes, Hcy may also play a role in the development of AD by antagonizing gamma-aminobutyric acid (GABA) receptors and acting as a neurotransmitter competing with GABA [110,111,112]. In addition, Hcy has been implicated in the pathogenesis of other neurological and psychiatric diseases in humans, including autism, epilepsy, depression, bipolar disorder and schizophrenia [31,113,114,115,116].

There is no data on the role of Hcy in equine neurological diseases. However, the involvement of Hcy in Parkinson’s disease (PD), which is pathogenetically similar to equine pituitary pars intermedia dysfunction (PPID) [117], provides an exciting area for future research into the role of Hcy in this disease entity in horses. Other potential research avenues include the investigation of the role of Hcy in equine degenerative myeloencephalopathy (EDM) and equine motor neuron disease (EMND) [118,119].

### 4.3. Physical Activity

Horses are exceptionally well-adapted athletes [120]. This adaptative behaviour has been crucial in the species’ survival in the face of the threat from predators [121]. During exercise, a horse’s heart rate increases by more than eight times concerning resting conditions [122]. Additional adaptative manifestations to increased physical load include an increased ratio of lung capacity to body weight, the ability to double the number of peripheral blood erythrocytes by splenic contraction in the initial phase of physical effort, and even adaptation to hypercapnia under extreme exercise load [122,123,124].

Nevertheless, despite the adaptation mechanisms, sports horses are subjected to adverse effects of physical exertion. Apart from injuries, myopathies, post-exercise pulmonary haemorrhage and other clinical conditions, strenuous exercise and increased oxygen consumption generate oxidative stress, and free radical formation is responsible for numerous metabolic disorders [125,126,127,128]. Furthermore, in humans, strenuous exercise has been linked to cardiac injury by the induction of oxidative stress and systemic inflammatory response [129].

It has been demonstrated that acute exercise increases Hcy levels in humans, and the magnitude of those elevations depends on prior training preparation [130,131]. Although the basal Hcy serum concentration is lower in people with regular physical activity, up to 47% of athletes develop hyperhomocysteinemia after exercise [132,133]. It raises concerns for amateur runners participating in triathlons, marathons or ultramarathons. Prolonged post-exercise oxidative stress and hyperhomocysteinemia may lead to vascular endothelial damage in these individuals [131].

In one of the few studies evaluating the relationship between Hcy and strenuous exercise in horses, animals were subjected to jumping over ten obstacles with a maximum height of 140 cm over a distance of 350 m [60]. The serum Hcy concentrations were obtained before, 30 and 60 min after the training, with a tendency for increased Hcy concentration documented immediately after training [60].

The influence of strenuous exercise on the levels of antioxidants and vitamins is significant as an increased breakdown of glycogen and an increased demand for B6 post-training is well described [134]. Interestingly, post-exercise increase in serum Hcy levels has been detected in humans with appropriate concentrations of folic acid and vitamin B6 [131]. Hyperhomocysteinemia in those subjects has been explained by natural post-exercise changes in glomerular filtration and plasma clearance of Hcy. None of those relationships has been studied in horses.

Physical exercise can cause various biochemical changes that may affect horses’ oxidative stress-dependent Hcy metabolic pathways. It has therefore been suggested to study the physiological range of serum Hcy and its changes depending on the frequency, intensity and duration of training [60].

### 4.4. Oxidative Stress

Hcy is an important inducer of prooxidative-antioxidative imbalance associated with increased intracellular calcium ion concentrations and DNA damage [98,135]. Hcy contributes to oxidative stress by reducing glutathione peroxidase activity and lowering the level of vitamins A, E and C [136]. By reducing the de novo synthesis of glutathione, Hcy also leads to the impairment of redox mechanisms [137]. Stimulation of the synthesis of reactive oxygen species is one of the most important links between Hcy, particularly its oxidized sulfhydryl groups, and the development of atherosclerosis [138]. Atherosclerosis is also favoured by Hcy-induced hyperuricemia, which further promotes the formation of reactive oxygen species and subsequent vascular endothelium dysfunction [85].

Oxidative stress contributes to increased uptake and use of methionine, which is subsequently converted to Hcy. It has been demonstrated that the maximal physical activity of racehorses over 1200, 1600 and 2000 m causes an increase in reactive oxygen metabolites (dROMs) and serum Hcy concentration. The mentioned indices remained positively correlated with each other also after strenuous physical activity [63].

In another study examining the relationship between Hcy and oxidative stress in racing horses, there was a high positive correlation between the serum concentration of Hcy and dROMs. The Hcy concentration increased immediately after exercise, returned to resting values 30 min later, and remained stable until the end of observation (i.e., 180 min after the race). The concentration of dROMs initially increased immediately after exercise, then decreased. However, it remained higher concerning the resting value for the remainder of the observation period. Post-exercise increases and decreases in serum Hcy concentration were also positively correlated with the antioxidative barrier (Oxy-Adsorbent), a compensatory response to oxidative stress [59].

The effects of oxidative stress on serum biomarkers have already been identified in racehorses, where an elevation in gamma-glutamyl transferase (GGT) associated with training has been documented [139]. A relationship between poor performance and elevated gamma-glutamyl transferase (GGT) beyond this anticipated increase has also been identified. Although the exact mechanism responsible for the increase in GGT is not fully understood, a poor response to oxidative stress (overtraining) has been proposed [139]. A recent investigation to determine the potential role of infectious agents with elevated GGT in a population of racehorses also supports the role of oxidative stress and, in addition, identified a decrease in vitamin B6 in the horses with exercise-associated elevation in GGT [140]. These data further support the investigation of Hcy in racehorses, particularly in a subset of horses with poor performance and elevated GGT.

Those findings suggest that sports horses may be at risk of certain Hcy-related diseases and, at the same time, provide the rationale for serum Hcy as a potential marker of redox disorders in this species.

### 4.5. Reference Values and Hyperhomocysteinemia

Hyperhomocysteinemia in humans is defined as serum Hcy concentration exceeding 15 µmol/L, while an increase of 2.5 µmol/L above this value increases the risk of cardiovascular disease by 10% [141,142].

Currently, it is difficult to clearly define hyperhomocysteinemia in horses due to a lack of established and standardized reference ranges for serum Hcy and a limited number of studies on the role of this amino acid in disease pathogenesis. However, under physiological conditions in horses, elevated post-training serum Hcy levels are usually short-lived, lasting for around 60 min post-exercise [60]. Furthermore, some evidence suggests that the length of post-exercise serum Hcy elevation in horses is directly proportional to the length of prior strenuous exercise [131].

To the best of our knowledge, only three studies assessed normal serum Hcy concentration in horses. A study in ponies reported normal serum Hcy concentration in the range of 1.3–14.7 µmol/L [58]. Another small research estimated average resting serum Hcy at 6.16 µmol/L (SD 0.36), with interindividual variations likely caused by differences in husbandry factors [60]. In a research of a more diverse group of 27 healthy horses, the serum Hcy reference range was established at 1.5–7.8 µmol/L with an average value of 4.65 µmol/L [61]. More research is needed to reliably show the physiological range of serum Hcy and its normal variation in horses.

### 4.6. Determination Methods

Serum Hcy concentration has been successfully and reproducibly determined in dogs by chemiluminescent immunoassay incorporated in the ADVIA Centaur XP automated system (Siemens, Munich, Germany) [42]. The correlation between the results obtained by this method and the results obtained by high-performance liquid chromatography (HPLC) is almost complete (r = 0.96, *p* = 0.0001) [143]. Similarly, an automated enzymatic method (Homocysteine Cobas C INTEGRA 800 assay, Roche Diagnostics International Ltd., Rotkreuz, Switzerland) has been reliably used to determine serum Hcy in horses [61]. Unlike previously utilised HPLC, both methods are rapid and easily available.

## 5. Conclusions

The involvement of Hcy in numerous pathogenetic processes in humans and animals provides a solid ground for further research in horses. Investigations into the role of Hcy in various diseases and physiological conditions, including physical exertion of varying intensity, are warranted. Most importantly, however, developing a standardized, unified and reliable reference range of serum Hcy in healthy horses is crucial. After meeting these criteria, serum Hcy will likely serve as a clinically useful surrogate marker for evaluating redox disorders in this species.

## Figures and Tables

**Table 1:** Summary of studies (chronologically) on the role of Hcy in the disease pathogenesis in horses.

Keywords	Publication Date	Reference
Embryonic resorption; mare	2003	[57]
Vascular endothelium; laminitis	2004	[58]
Reactive oxidant species (dROMs); antioxidant barrier (Oxy-adsorbent); thiol antioxidant barrier (SHp)	2009	[59]
Acute exercise; workload; serum lactate	2010	[60]
Cardiovascular; arrythmia; atrial fibrillation	2018	[61]
Cardio-renal biomarkers; parasitemia; *Theileria equi*	2020	[62]
Aspartate aminotransferase; creatine kinase; lactate dehydrogenase; oxidative stress	2022	[63]

## Data Availability

Data sharing is not applicable to this article.
